# Short-term effects of intravitreal ranibizumab therapy on diabetic macular edema

**DOI:** 10.1186/s12886-017-0420-8

**Published:** 2017-03-14

**Authors:** Yoshiro Minami, Taiji Nagaoka, Akihiro Ishibazawa, Akitoshi Yoshida

**Affiliations:** 1 Department of Ophthalmology, Nayoro City General Hospital, Nishi 7 Minami 8-1, Nayoro, 096-8511 Japan; 2 Department of Ophthalmology, Asahikawa Medical University, Asahikawa, Japan

**Keywords:** Diabetic macular edema, Retina, Ranibizumab, Optic coherence tomography

## Abstract

**Background:**

The short-term effects of intravitreal ranibizumab (IVR) on diabetic macular edema (DME) remains unclear. We assessed the short-term effects of IVR on DME.

**Methods:**

Eighteen eyes of 14 patients with DME were enrolled in this prospective interventional case series. After intravitreal ranibizumab was injected into treatment-naïve eyes with DME, we measured the foveal thickness (FT) before and 2 h, 1 day, 1 week, and 1 month later and the best-corrected visual acuity (BCVA) at all times except 2 h and compared the changes to baseline (ΔFT and ΔVA).

**Results:**

The mean FT decreased significantly (*p* < 0.0001) from 452 ± 77 to 429 ± 65 microns after 2 h. The mean logarithm of the minimum angle of resolution BCVA improved significantly (*p* = 0.032) after 1 month from 0.41 ± 0.24 to 0.32 ± 0.21 (20/51 to 20/42, Snellen equivalent). The ΔFT after 2 h was significantly (*r* = 0.53, *p* = 0.025) correlated with the ΔFT after 1 month. The ΔVA after 1 day was significantly (*r* = 0.59, *p* = 0.01) correlated with the ΔVA after 1 month.

**Conclusions:**

The structural effects of IVR for DME occurred within 2 h, whereas the functional effects occurred after 1 month. The short-term effects (within 1 day) of IVR may predict the therapeutic outcome 1 month after IVR in patients with DME.

**Trial registration:**

The trial registration number: UMIN000026118 (Feb/13/2017). Retrospectively registered.

## Background

Diabetic macular edema (DME) is the major cause of visual loss in working age patients in developed countries [1]. The efficacy of intravitreal ranibizumab (IVR) (Lucentis, Novartis, Basel, Switzerland), a humanized affinity-matured vascular endothelial growth factor (VEGF) antibody fragment that specifically binds all isoforms of VEGF-A as a therapy for DME is now well established. Multiple randomized, controlled clinical trials, including the DRCR.net Protocol I [2, 3], RIDE and RISE [4, 5], READ 2 [6, 7], RESOLVE [8], RESTORE [9, 10], and REVEAL [11], have demonstrated visual improvement with IVR therapy. In these clinical trials, the first evaluations of the therapeutic effect on the foveal thickness (FT) and visual acuity (VA) were performed 1 week [8] or 1 month [2–7, 9–11] after injection of ranibizumab. To our knowledge, the short-term effects, i.e., within a few hours, after IVR injection on DME have not been evaluated. In the current study, we determined if the short-term effects (within 1 day) of IVR injection might predict the long-term (1 month) outcomes of the FT and VA in patients with DME.

## Methods

### Subjects

The study adhered to the tenets of the Declaration of Helsinki and followed the guidelines approved by the ethics committee of our institution. All patients were native Japanese who provided informed consent before participation in the study. Twenty-four consecutive eyes of 20 patients with DME were enrolled and treated prospectively with ranibizumab from April 2014 to May 2015. The inclusion criteria were the presence of DME before therapy (FT at baseline ≧ 300 microns) and no history of ocular surgery (including laser) and/or other treatment for macular edema within the previous 10 weeks. No patients were treated with a dexamethasone implant because the treatment has not yet been approved in Japan. No patients had a history of intravitreal anti-VEGF therapy. The exclusion criteria were the logarithm of the minimum angle of resolution (logMAR) VA below 0 (20/200, Snellen equivalent). The patients underwent comprehensive ophthalmologic examinations including measurement of the best-corrected VA (BCVA), slit-lamp biomicroscopy with a noncontact fundus lens, and spectral-domain optical coherence tomography (SD-OCT) (RetinaScan RS-3000, Nidek, Gamagori, Japan). The BCVA was measured using a standard Japanese decimal VA chart at 5 m. The decimal values were converted to logMAR units for statistical analyses. To evaluate the FT, the macular map analysis protocol of the RS-3000 SD-OCT was used. The FT was defined as the average of all points in the inner circle (radius of 1 mm) at the center of the nine sectors defined by the Early Treatment Diabetic Retinopathy Study grid [12].

### IVR injection

IVR was administered in a sterile manner (0.5 mg/0.05 mL) using a 30-gauge needle. Before injection, anterior chamber paracentesis was performed using a 27-gauge needle to prevent intraocular pressure increases. Topical antibiotics were applied prophylactically for 1 week after the IVR injection.

### Time course of the evaluation of the therapeutic effect of IVR

The FT was measured before the IVR injection (baseline) and 2 h, 1 day, 1 week, and 1 month later. The BCVA was measured at the same times except for 2 h after the IVR injection. The changes in the FT (ΔFT) (in microns) from baseline to each time point were calculated and defined as the changes at 2 h, 1 day, 1 week, and 1 month (ΔFT-2 h, −1d, −1w, and -1 m, respectively). The changes in the logMAR VA (ΔVA) from baseline also were calculated as ΔVA-1d, −1w, and -1 m, respectively.

### Data analysis

All values are expressed as the mean ± standard deviation. We confirmed that the FTs and VAs at every time point were normally distributed using the D’Agostino-Pearson test. The overall differences in the FT at baseline, 2 h, 1 day, 1 week, and 1 month after IVR injection and in the logMAR VA at baseline, 1 day, 1 week, and 1 month after IVR injection were assessed using repeated measures analysis of variance (ANOVA) following Dunnett’s multiple comparisons test. To investigate if the early effectiveness of an IVR injection predicts the late-phase outcome after an IVR injection, the correlations between the ΔFT-2 h and the ΔFT-1 m were evaluated using Pearson’s correlation model and linear regression analysis. The correlations between the ΔVA-1d and ΔVA-1 m also were evaluated using Pearson’s correlation model and linear regression analysis. *p* < 0.05 was considered significant.

## Results

One patient withdrew from the current study for personal reasons. Five eyes of five patients were excluded; three patients underwent photocoagulation therapy within 2 months and two patients had a VA lower than 20/200 Snellen equivalent. Eighteen eyes of 14 patients were eligible in inclusion in this study. Fourteen eyes had non-proliferative diabetic retinopathy, and four eyes had proliferative diabetic retinopathy treated with panretinal photocoagulation over 3 months before entry into the current study. The average hemoglobin A1c value was 6.8%. Table 1 shows the patient baseline characteristics.

**Table 1:** Baseline characteristics of patients with diabetic macular edema

Age, years (mean ± SD)	62.9 ± 11.9
Men/women	8/6
Duration of diabetes (months, mean ± SD)	10.2 ± 7.6
DR scale (eyes)	NPDR 14
	PDR 4
Hemoglobin A1c (%)	6.8 ± 0.5
History of photocoagulation (eyes)	14 (57%)
History of cataract surgery (eyes)	6 (33%)
VA (logMAR, mean ± SD)	0.41 ± 0.24
Baseline FT (microns, mean ± SD)	452 ± 77

*NPDR* non-proliferative diabetic retinopathy, *PDR* proliferative diabetic retinopathy, *logMAR* logarithm of the minimum angle of resolution, *SD* standard deviation, *VA* visual acuity, *FT* foveal thickness

**Fig. 1:**
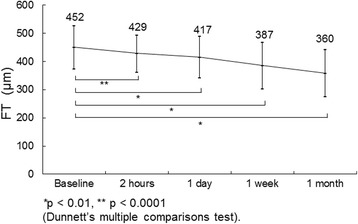
The mean changes in foveal thickness (FT) in 18 eyes at each follow-up time point. The values are expressed as the mean ± standard deviation. The FT is decreased significantly (*p < 0.01, **p < 0.001, ***p < 0.0001) from baseline

All patients had type 2 diabetes mellitus. No treatment complications such as endophthalmitis or retinal detachment developed during the study. Fig. 1 shows the time course of the mean changes in the FT after IVR injection. The mean FT decreased significantly (*p* < 0.0001) from the baseline value of 452 ± 77 microns to 429 ± 65 microns 2 h after the IVR injection. Significant (*p* = 0.002, *p* = 0.002, *p* = 0.0005) reductions also were seen to 417 ± 74, 387 ± 83, and 360 ± 84 microns on 1 day, 1 week, and 1 month, respectively.

**Fig. 2:**
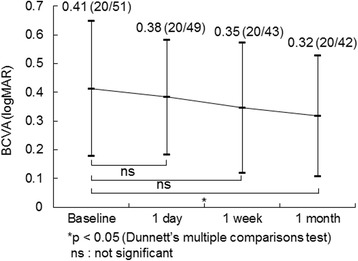
The mean changes in the logarithm of minimum angle of resolution (logMAR) best-corrected visual acuity (BCVA) at each follow-up evaluation in 18 eyes. The values are expressed as the mean ± standard deviation (Snellen equivalent). The BCVA is improved significantly from baseline (*p < 0.05)

Figure 2 shows the mean changes in the logMAR VA after IVR injection. The mean logMAR VA improved significantly (*p* = 0.032) from the baseline 0.41 ± 0.24 (20/51, Snellen equivalent) to 0.32 ± 0.21 (20/42, Snellen equivalent) 1 month after IVR injection. The logMAR VAs at 1 day (0.38 ± 0.20) and 1 week (0.35 ± 0.23) (20/49 and 20/43, Snellen equivalent) after IVR injection did not improve significantly (*p* = 0.62 and *p* = 0.12, respectively) from baseline.

**Fig. 3:**
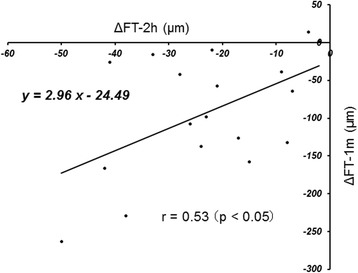
The relationship between the changes in the foveal thickness (FT) from baseline to 2 h (ΔFT-2 h) and the changes in FT from baseline to 1 month (ΔFT-1 m) after intravitreal ranibizumab injection. There is a significant (*r* = 0.53, *p* < 0.05) positive correlation between them

**Fig. 4:**
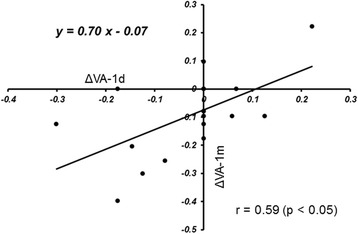
The relationship between changes in the logarithm of the minimum angle of resolution (logMAR) best-corrected visual acuity (BCVA) from baseline to 1 day (ΔVA-1d) and the changes in the BCVA from baseline to 1 month (ΔVA-1 m) after injection. There is a significant (*r* = 0.59, *p* < 0.05) positive correlation between them

**Fig. 5:**
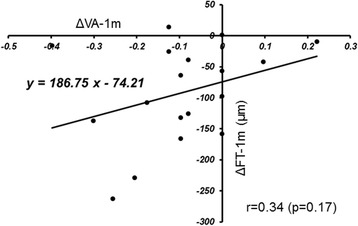
The relationship between the changes in the foveal thickness (FT) from baseline to 1 month (ΔFT-1 m) and the changes in the logarithm of the minimum angle of resolution (logMAR) best-corrected visual acuity (BCVA) from baseline to 1 month after injection. There is no significant (r = 0.34, *p* = 0.17) correlation between them

The ΔFT-2 h was correlated significantly (*r* = 0.53, *p* = 0.025) with the ΔFT-1 m (Fig. 3). The ΔVA-1d was correlated significantly (*r* = 0.59, *p* = 0.01) with the ΔVA-1 m (Fig. 4). There was no significant (*r* = 0.34, *p* = 0.17) correlation between the ΔFT-1 m and ΔVA-1 m (Fig. 5).

There was no significant (*p* = 0.06) correlation between the baseline BCVA and the ΔVA-1 m. The baseline BCVA was significantly (*p* < 0.0001) correlated with the BCVA at 1 month The baseline FT was significantly (*p* < 0.003) correlated with the FT at 1 month.

## Discussion

The current study showed that the FT decreased significantly 2 h after IVR injection in patients with DME. Welch et al. [13] previously reported that the FT decreased significantly 1 to 2 h after intravitreal injection of bevacizumab (IVB) (Avastin, Genentech Inc., South San Francisco, CA) in seven patients with DME and two patients with exudative age-related macular degeneration (AMD). Those investigators reported a significant decrease in OCT thickness within 2 h after injection. Although they used a different anti-VEGF drug (bevacizumab) in patients with DME and AMD, the results agree with the current findings.

We observed a significant positive correlation between the ΔFT-2 h and ΔFT-1 m (Fig. 3). The current results suggested that we can predict the FT 1 month after an IVR injection by measuring the FT as early as 2 h after the IVR injection. Unfortunately, the long-term effect of IVR remains unknown due to the current short follow-up period. Therefore, we could not conclude definitively if the short-term effects of an IVR injection is correlated with the long-term effects more than 1 month after an IVR injection administered to treat DME. Further study with a longer follow-up period is warranted to examine whether the long-term effects of an IVR injection can be predictable based on the short-term effects.

Moreover, there was a significant correlation between the ΔVA-1d and ΔVA-1 m (Fig. 4), suggesting that it is possible to predict the BCVA 1 month after treatment by measuring the BCVA 1 day after IVR injection. Ma et al. reported that the FT 1 h after IVB injection significantly decreased compared with baseline and that a reduction in the FT 1 h after IVB was correlated significantly with the reduction in the central macular thickness 1 month after IVB injection in patients with both DME and macular oedema after branch retinal vein occlusion (BRVO) (total of 30 eyes). The authors speculated that the FT 1 month after treatment might be predictable by measuring it a few hours after IVB injection [14].

We found a significant correlation between the baseline FT and the FT at 1 month. It was reported that the baseline FT might predict the structural outcomes in response to IVR therapy [15]. There also was a significant correlation between the baseline BCVA and the BCVA at 1 month. As previously reported, the baseline BCVA might predict the functional outcome after IVR therapy [9, 11]. Taken together, we speculated that measuring the efficacy as early as 1 day after an IVR injection in patients with DME might be predictive of the structural and functional effects of the IVR injection in addition to the prediction from the baseline FT and BCVA. In contrast, there was no significant (*p* = 0.06) correlation between the baseline BCVA and the ΔVA-1 m. However, eyes with a low baseline VA tended to have a large increase in the ΔVA-1 m in the current study as previously reported [16].

Previous major clinical trials have reported that the VA improvements from baseline tended to be associated with reductions in the FT from baseline [2, 3, 9, 10, 17]. Indeed, there was a significant (*r* = 0.48, *p* < 0.05) correlation between the ΔFT-1w and ΔVA-1w in the current study. Another clinical study with a large number of patients is needed to confirm the correlation between the improvements in BCVA and improvements in FT.

Pro re nata (PRN) regimens guided by VA have been reported to be effective for treating DME [3, 10, 18, 19]. However, patients might be undertreated based on OCT findings in treat-and-extend protocols [20]. In order to choose adequate injection regimens, the ability to gauge the required treatment intensity might be helpful. The results of the current study indicated that the short-time change could help with this and predict the long-term response.

We recently determined the efficacy of IVR injections in patients with macular edema due to BRVO [21]. In BRVO, the FT decreased significantly at 2 h, 1 day, 1 week, and 1 month after IVR injections, the same as in the current report. Although the baseline FT differed (522 ± 131 μm vs 452 ± 77 μm, BRVO vs DME, *p* = 0.049), the ΔFTs were significantly higher in BRVO compared with DME at 1 day, 1 week, and 1 month after IVR injections if the ΔFT was divided by the baseline FT (*p* < 0.001, by the two-way ANOVA and Sidak multiple comparisons test) (data not shown). We speculated that those differences in the efficacy of IVR injection between DME and BRVO might have resulted from the differences in the mechanisms of the macular edema. Campochiaro et al. reported that several cytokine levels in the aqueous humor differed between DME and the macular edema after BRVO [22]. Further clinical studies including additional measurement of the intraocular cytokine levels in eyes treated with IVR injections are needed to clarify the differences between DME and macular edema after BRVO. We also believe that measuring the short-term effects of IVR injection is useful not only to predict the efficacy but also to consider the difference in the mechanisms of macular edema between DME and BRVO.

The current study had some limitations. First, the number of patients in this case series was too small to perform a subgroup analysis. Another larger clinical study is needed. Second, the current follow-up period was short and another clinical study with a long follow-up period is necessary to determine whether or not the short-term effects of an IVR injection on the BCVA and FT are correlated with the long-term effects of the IVR injection on the BCVA. Third, the current study had no control group. We could not exclude the influences of the natural disease course or the effects of previous treatments more than 10 weeks before IVR injection on the current results. It was reported that retinal thickness measurements vary over the course of a day [23]. In the current study, we could not evaluate the effect of circadian fluctuation and the reproducibility and variations in the retinal thickness measurements in both healthy subjects and patients. Another clinical study that includes a control group and repeated measurements is needed.

## Conclusion

In conclusion, the current findings suggested that the structural effects of IVR injections might be detectable as early as 1 day after treatment. We believe that evaluation of the short-term effects of IVR injections can predict the therapeutic outcome 1 month after IVR injection for DME.

## Abbreviations

BCVA: Best-corrected VA
BRVO: Branch retinal vein occlusion
DME: Diabetic macular edema
FT: Foveal thickness
IVB: Intravitreal injection of bevacizumab
IVR: Intravitreal ranibizumab
logMAR: Logarithm of the minimum angle of resolution
MD: Age-related macular degeneration
PRN: Pro re nata
SD-OCT: Spectral-domain optical coherence tomography
VA: Visual acuity
VEGF: Vascular endothelial growth factor
ΔFT: Changes in the FT
ΔVA: Changes in the logMAR VA
